# A long-lasting emerging epidemic of anthroponotic cutaneous leishmaniasis in southeastern Iran: population movement and peri-urban settlements as a major risk factor

**DOI:** 10.1186/s13071-021-04619-3

**Published:** 2021-02-24

**Authors:** Taiebeh Karimi, Iraj Sharifi, Mohammad Reza Aflatoonian, Behnaz Aflatoonian, Mohammad Ali Mohammadi, Ehsan Salarkia, Zahra Babaei, Farzaneh Zarinkar, Fatemeh Sharifi, Nima Hatami, Ahmad Khosravi, Arsalan Eskandari, Elyas Solimani, Mehdi Shafiee, Masoumeh Mozaffari, Amireh Heshmatkhah, Rezvan Amiri, Saeideh Farajzadeh, Alireza Kyhani, Abbas Aghaei Afshar, Abdollah Jafarzadeh, Mehdi Bamorovat

**Affiliations:** 1 Department of Medical Parasitology and Mycology, Kerman University of Medical Sciences, Kerman, Iran; 2 Leishmaniasis Research Center, Kerman University of Medical Sciences, Kerman, Iran; 3 Research Center for Tropical and Infectious Diseases, Kerman University of Medical Sciences, Kerman, Iran; 4 Research Center for Hydatid Disease in Iran, Kerman University of Medical Sciences, Kerman, Iran; 5 Pharmaceutics Research Center, Institute of Neuropharmacology, Kerman University of Medical Sciences, Kerman, Iran; 6 Department of Endodontic, Faculty of Dentistry, Kerman University of Medical Sciences, Kerman, Iran; 7 County Health System Services, Kerman University of Medical Sciences, Kerman, Iran; 8 Provincial Health System Services, Kerman University of Medical Sciences, Kerman, Iran; 9 Shahid Dadbin Clinic, Kerman University of Medical Sciences, Kerman, Iran; 10 Department of Dermatology, Afzalipour Hospital, Kerman University of Medical Sciences, Kerman, Iran

**Keywords:** Population movement, Cutaneous leishmaniasis epidemic, PCR-RFLP, Phylogenetic analysis, Iran

## Abstract

**Background:**

Epidemics of cutaneous leishmaniasis (CL) are occurring more frequently and spreading faster and farther than before in many areas of the world. The present study aimed to assess a long-lasting emerging epidemic (2005–2019) of 5532 cases with anthroponotic CL (ACL) in peri-urban areas of Kerman city in southeastern Iran.

**Methods:**

This descriptive-analytical study was carried out for 15 years in Kerman province, southeastern Iran. The data were passively obtained through the health surveillance system and the Kerman Leishmaniasis Research Center. Every subject was diagnosed using direct smear microscopy. The representative causative agent was further examined by ITS1-PCR, PCR-RFLP, 7SL RNA gene sequencing and phylogenetic analyses. For each subject, a case report form designating demographic and clinical data was recorded.

**Results:**

A different pattern of ACL incidence was found in peri-urban areas compared to that in the city of Kerman. The incidence rate of ACL cases has significantly increased (*P* < 0.001) from 2005 to 2016 in new settlements with a gradual decline after that. The overall average risk of contracting the disease was 7.6 times higher in peri-urban areas compared to Kerman city, an old endemic focus. All isolates consisting of six variants were confirmed to be *Leishmania tropica*. The overall pattern of the ACL infection indicates that the etiological agent of ACL is propagated and transmitted by the bite of female *Phlebotomus sergenti* sandflies from person to person from dissimilar clones as reflected by the complexity of the migrants’ backgrounds in the province.

**Conclusions:**

The movement of populations and establishment of new settlements in peri-urban areas close to endemic areas are major risk factors for and are directly linked to CL. The underlying factors of this emerging ACL epidemic caused by *L. tropica* were disasters and droughts, among others. A robust commitment to a multilateral approach is crucial to make improvements in this area. This will require decisive coordinated actions through all governmental factions and non-governmental organizations. Furthermore, active and passive case detection strategies, early diagnosis, and effective treatment could help control the disease. 
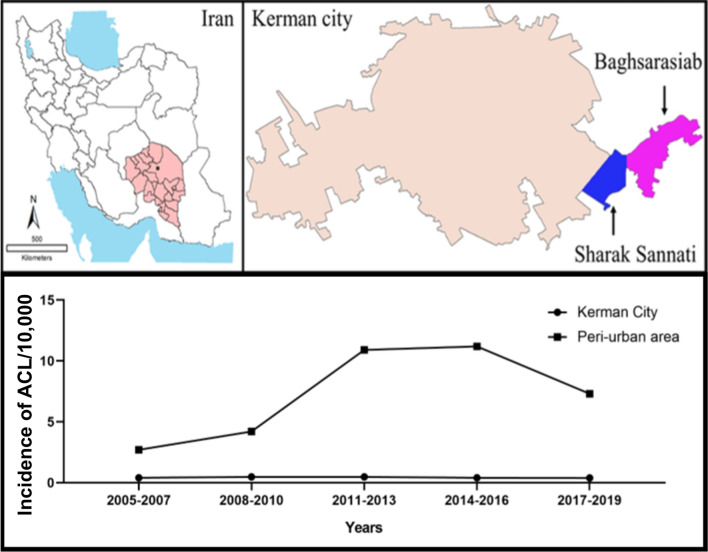

## Background

Leishmaniasis remains a serious concern among the most neglected and vector-borne diseases worldwide [1, 2]. There are several forms of classical and atypical leishmaniasis, among which three forms are principal: cutaneous leishmaniasis (CL), mucocutaneous leishmaniasis (MCL), and visceral leishmaniasis (VL) [3]. The disease burden is not well known, but based on recent estimates, up to 1.3 million new cases of leishmaniasis, 12 million prevalence, and some 26,000–65,000 deaths in 101 countries and territories occur annually among a population of 1 billion at-risk individuals [3, 4]. However, the current actual burden is much greater than reported in the previous statistics. Due to confounding factors, ongoing conflict, consequent migration, and unprecedented environmental risks, new CL figures propose that this disease is a large-scale social and medical problem in the affected countries [5]. Etiological agents involve a digenetic life-cycle between an invertebrate vector, phlebotomine sandflies, and a vertebrate reservoir host, such as humans and suitable animals [6].

Cutaneous leishmaniasis is the most epidemic-prone disease, a common type, and a widely dispersed form with serious public health complications in 70 countries where nearly 95% of CL cases occur, mainly in the world's tropics, namely the Americas, Mediterranean countries, the Middle East, and Central Asia. Predictably, the WHO Eastern Mediterranean Region alone accounts for 70% of the new cases globally, and the remaining cases (30%) occur in other parts of the world. In 2017, it was reported that > 95% of the incidence occurred in Afghanistan, Syria, Algeria, Iran, Colombia, and Brazil [3]. Cutaneous leishmaniasis produces cutaneous lesions on naked areas of the body, which are often exposed to the bite of female sandflies. The disease is self-healing but causes life-long cutaneous scars, prominent disfigurement, severe disability, serious depression, and social stigmatization [1].

Currently, no approved and efficacious vaccine against leishmaniasis is available. The use of conventional drugs is not sufficient, although liposomal amphotericin B has previously been recommended [7]. Moreover, there is an effective personal control measure for leishmaniasis. In a village-based cohort study, Olyset^®^ Plus, a novel long-lasting insecticidal net (LLIN), impregnated with 2% permethrin and piperonyl butoxide, was used against CL transmission. The protective efficacy level of LLIN was assessed to be 92.2% following 6 and 12 months of usage. The authors concluded that Olyset^®^ Plus nets are an effective and durable personal protection measure to prevent sandfly bites in a hyperendemic area of Turkey [8].

The recent unprecedented national and global spread of CL has highlighted the challenges faced by many countries. Most vector-borne diseases (VBDs) such as CL can be prevented through vector control, but only if implemented effectively. This is, however, hampered by numerous challenges that include: lack of capacity and capability in programs, lack of a comprehensive national strategy, the absence of a legal framework for a vector control intervention toolbox, lack of community involvement, and ongoing environmental and social changes that result in the proliferation and geographical expansion of vectors [9]. However, the potential risk for the *Leishmania* species in the Mediterranean region is variable and reported predominantly in the June-October period. Therefore, the highest protective effect will be attained between 11 p.m. and 2 a.m., coinciding with the nocturnal pattern of female sandfly activity [10].

Global studies indicated numerous emerging CL outbreaks over the past years in different parts of the world, including Peru [11], Brazil [12, 13], Afghanistan [14], Libya [15], Syria [16], Iraq [17], Yemen [18], Sudan [19], Morocco [20], and Saudi Arabia [21]. The CL situation is probably similar in the countries neighboring Iran. Due to the ongoing civil war in Syria, many Syrians have fled to Turkey and other neighboring countries, contributing to a drastic increase in CL cases predominantly caused by *L. tropica* [22]. Turkey is a crossroad between the Asian countries and Europe. A recent survey by Özden and colleagues indicated that approximately 4 million Syrian refugees arrived in Turkey before 2013 [23, 24]. Millions of other Syrians crossed the border into Turkey, causing serious socioeconomic consequences and severe public impacts in this country, and then moved toward Europe [25]. Despite the relative reduction in VL patients, the number of CL cases has been rising, mainly because of escalation of transmission in rural communities and suburban areas of the cities and the deterioration of economic and social conditions in highly exposed populations in the world [6].

In Iran, two CL types, including anthroponotic CL (ACL) and zoonotic CL (ZCL), are induced by *L. tropica* and *L. major*, respectively [26]. Anthroponotic cutaneous leishmaniasis is restricted to the Old World [27]. The parasite *L. tropica* is principally transmitted by *Phlebotomus sergenti*. Although CL is distributed in 17 of the 31 provinces in Iran, about 80% of the total cases belong to ZCL, and the remaining cases are believed to be ACL [26, 28]. The latter is mainly urban and peri-urban and displays patterns of spatial clustering in many localities. The disease is generally characterized by large epidemics in heavily populated cities. Major foci of ACL are in southeastern parts of the country (Kerman and Bam counties), Yazd, Mashhad, and, to a smaller extent, in other provinces of Iran [26]. This focal dispersion of CL is merely in micro-ecological niches that affect the etiological agent, the reservoir host, and the primary vector [29]. Kerman province, where over 95% of the cases are caused by *L. tropica*, represents a major focus of ACL in Iran [30]. This disease represents an emergent threat with a high social and public health burden in the country. Over the past decades, several emerging ACL and ZCL epidemics of variable magnitudes [31–35] have occurred in different provinces of Iran. Migration, low economic status, natural disasters (drought, earthquakes, and flood), poverty-stricken suburbs of large cities, and cross-border movements are distinct risk factors for the emerging ACL epidemics [27, 36, 37].

The association between war and the emergence of CL is notably illustratted in Middle Eastern countries. During these wars, a wide range of risk-associated determinants such as population movement, the formation of new settlements, ecological disturbances, the collapse of control measures, and the breakdown of the health surveillance system have played a crucial role in the drastically increased incidence of CL in Syria, Saudi Arabia, Afghanistan, Lebanon, Libya, Iraq, and Turkey [24]. Currently, the disease has reached epidemic levels, affecting refugees in the involved zones. Of notable concern is CL’s long-term burden on conflict-ridden and previously endemic countries and particularly the role of CL in promoting severe poverty in such situations. As a result, the disease is now a large-scale global health concern. These outcomes have important health implications relevant to the prioritization of CL in global disease control strategies and research and development (R&D) needs, including diagnostic tools, drug innovations, and vaccine development [5].

This study aimed to assess an emerging epidemic focus of ACL in peri-urban areas of the city of Kerman in southeastern Iran. This focus has gradually been established, mainly because of the population movement and creation of new settlements. This investigation was undertaken because of political concerns, frequent earthquakes, and drought. Herein, the epidemiological, molecular, and phylogenetic profiles and associated public health impacts of the disease are explored.

## Methods

### Ethical considerations

The project (No. 92.448 and Ethics Code IR.KMU.REC.1392.413) was approved by the Ethics Committee of the Institutional Review Board (IRB) of Kerman University of Medical Sciences. The suspected CL cases were referred to the CL control clinic for diagnosis and free treatment. A brief face-to-face educational training was planned for each patient at the clinic to familiarize them with the disease consequences, proper management, and possible future relapses. Those who had underlying complications were referred to higher hospital levels to be further examined and managed accordingly. The patients only received a routine diagnosis and medication free of charge. The main reason why oral consent was acquired instead of written agreement was the illiteracy of most participants. No attempt was made to obtain written informed consent because of the possibility of the participant’s refusal to participate.

### Study area

**Fig. 1:**
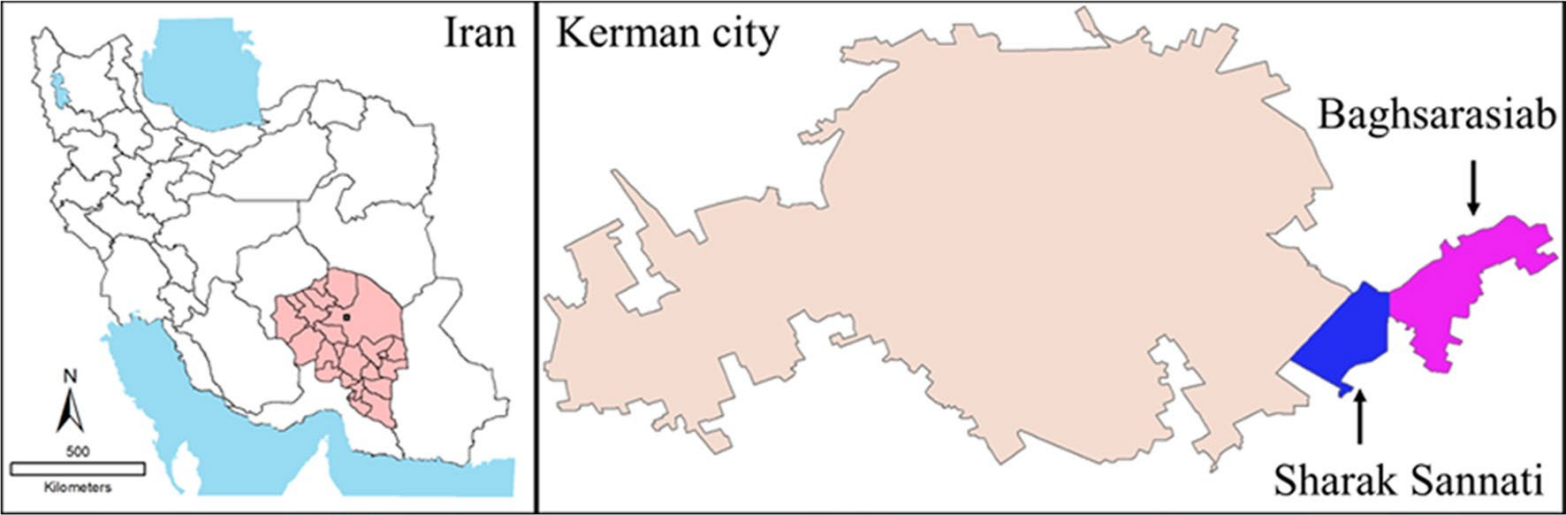
The affected peri-urban areas located in the southeastern part of Kerman city, southeastern Iran

The province of Kerman is located between 30°16'59.56"N and 57°4'43.64"E and is the largest province in Iran with an area of 183,285 km^2^ and a population of 3,200,000. This province is located in the southeastern part of the country, 1000 km from Tehran (Fig. 1). The climate is diverse and directly influenced by two distinct types of climatic conditions: the southern areas include Bam, which is hot in summer (42 °C) and has a moderate climate in winter (25 °C), while the northern parts include Kerman city and the neighboring counties, with a mild and arid climate and a minimum and maximum temperature of − 7 °C and 40 °C, respectively. Such a varied range of climatic conditions creates suitable conditions for agricultural activities. This province alone supports approximately 25% of the country’s gardens. The above microclimatic zones and fertile land create appropriate conditions favoring CL proliferation.

The peri-urban area was home to 18,968 inhabitants in 2019 and consisted of two complex localities, Baghsarasiab, and Sharak Sannati (Table 1 and Fig. 1). Most migrants (80%) gradually arrived in this peri-urban area either from ACL endemic areas such as Bam and neighboring counties and non-endemic counties such as Jiroft, Zarand, and Sirjan.

**Table 1:** Population composition of the peri-urban area in the southeastern part of the city of Kerman, southeastern Iran, 2019

Origin	No. (%)
Indigenou (Baghsarasiab/Sharak Sannati)	3793 (20.0)
Migrants	15,175 (80.0)
Bam/neighboring counties	4469 (31.4)
Non-endemic counties	8403 (55.4)
Afghan	2003 (13.2)
Total	18968 (100.0)

The data were obtained through the health clinic stationed in the peri-urban area

### Data collection and sampling

This descriptive-analytical study was undertaken from July 2005 to December 2019 in the peri-urban area, and the data of the patients were compared with those obtained from the residents of the city of Kerman, the adjacent endemic city, where ACL has long been endemic. Pre-2005, data were not available as there was no well-recorded registry and referral system for CL patients to be documented and systematically managed. A general health clinic in charge of primary health care (PHC) services has previously been assigned in the peri-urban area (at the Sarasiab location). To determine this peri-urban area's population, a repeated census house-to-house visit is undertaken yearly by the health personnel. Subjects are passively referred to the health clinic to be screened for CL and other diseases. The peri-urban migrants who had resided in the area for at least 3 months were included. However, immigrants who left the peri-urban area within 3 months were excluded. Moreover, those settlers who had initial scars or a history of skin lesion(s) and were diagnosed with CL infection upon direct smear preparation were excluded from the study.

Every subject underwent a thorough physical examination of all body areas. The suspected cases were referred to Dadbin Health Clinic as the main registry center for managing CL cases. This health clinic has directly affiliated with the Leishmaniasis Research Center and School of Medicine. For each subject, a case report form (CRF) designating demographic (age, sex, and nationality) and clinical data (anatomical location and number of lesions, and treatment outcome) was recorded. In addition to direct smear preparation for all cases, clinical specimens were randomly selected for further molecular, sequencing, and phylogenetic analyses.

### Clinical samples

Clinical smears were prepared from the CL-positive cases using a scalpel and blade from the margin of each lesion. Smear preparations were dried, fixed, and routinely stained by Giemsa and examined for Leishman bodies (amastigotes) under a conventional optical microscope with high dry magnification (1000×) [38].

## Molecular identification

### DNA extraction

Altogether, 50 positive microscopic slides were randomly selected, cleaned by xylol, and placed into a 1-ml cell lysis buffer. Smear preparations were entirely scraped with a sterile scalpel and blade [39]. For identification of the causative species, the genomic DNA was extracted from previously prepared slides using DNeasy Blood and Tissue Kits (Qiagen, Germany) according to the manufacturer’s instruction. The extracted DNA was stored at − 20 °C for polymerase chain reaction (PCR) assays. Previously, over 900 isolates were identified to the species level from this peri-urban area and the city of Kerman [40–42].

### PCR analysis

Specimens were analyzed using the following forward and reverse ITS1-PCR primers as previously described with minor modifications: LITSR (5'-CTG-GAT-CAT-TTT-CCG-ATG-3') and L5.8S(5'-TGA-TAC-CAC-TTA-TCG-CAC-TT-3') [43, 44]. In brief, the thermal profile included three min at 94°C (primary denaturation), followed by 38 cycles of 30 s at 95 °C (denaturing), 35 s at 53 °C (annealing), and 45 s at 72 °C (extension), and a final extension step for 5 min at 72 °C. Polymerase chain reaction-specific amplification was confirmed by electrophoresing 5 µl of the reaction mix on 1% agarose gel containing ethidium bromide.

### RFLP analysis of the ITS_1_ PCR

Restriction fragment length polymorphism (RFLP) analysis was performed to exploit variations in DNA sequences for the 7SL RNA region to pinpoint the locations of genes with a specific sequence. PCR products were digested with HaeIII enzyme, based on the manufacturer’s protocol. The fragments were analyzed in 3% agarose gel by electrophoresis and visualized with ethidium bromide using UV light.

### Sequencing and phylogenetic analyses

Products of 18 randomly selected specimens were sequenced for the 7SL RNA region by Semiconductor Co., Ltd. Seoul, Korea. Sequences were trimmed by Sequence Scanner version 1 (Applied Biosystems Inc., Waltham, MA, USA). Multiple sequence alignments were analyzed using BioEdit program version 6. Molecular data were the basis for constructing the phylogenetic tree using MEGA version 7 [45].

### Statistical analysis

Data were analyzed using SPSS 22 (Chicago, IL, USA) with *χ*^2^ testing to evaluate the significance of the difference between proportions. Potential demographic and clinical characteristics for patients with ACL were assessed. The incidences (new cases) of ACL among different age groups, genders, nationalities, anatomical locations, number of lesions, and treatment outcome were evaluated for the peri-urban area and the city of Kerman. The odds ratio (OR) is the ratio of the new cases in the peri-urban area to those of the city of Kerman between 2005 and 2019. *P*-value < 0.05 was considered statistically significant.

## Results

### Epidemiological data

**Fig. 2:**
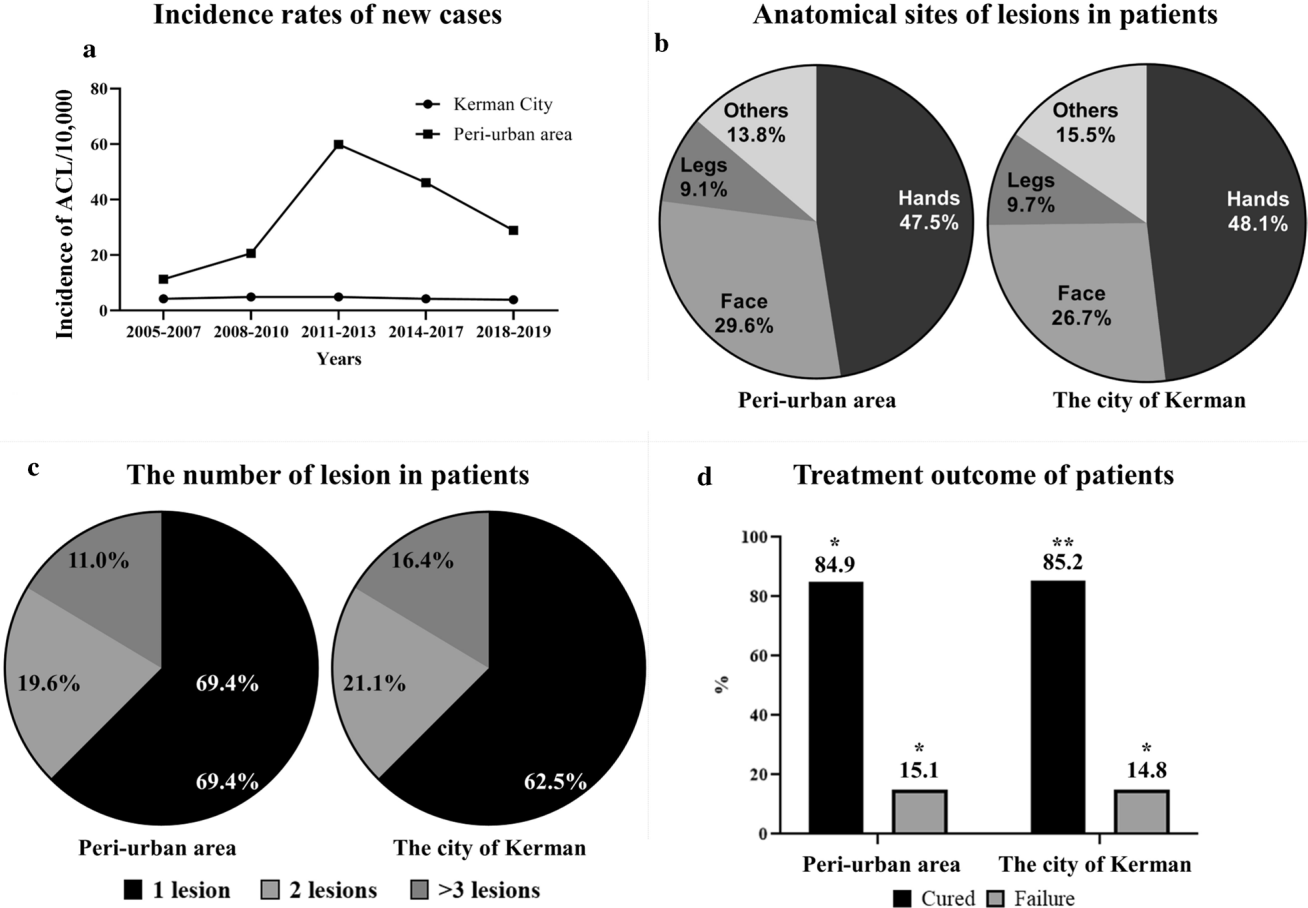
Incidence rates of new anthroponotic cutaneous leishmaniasis (ACL) cases (**a**), anatomical sites of the lesion in patients (**b**), number of lesions in patients (**c**) and treatment outcome of the patients (**d**) with ACL in the peri-urban area compared with the city of Kerman, southeastern Iran, 2005–2019

**Fig. 3:**
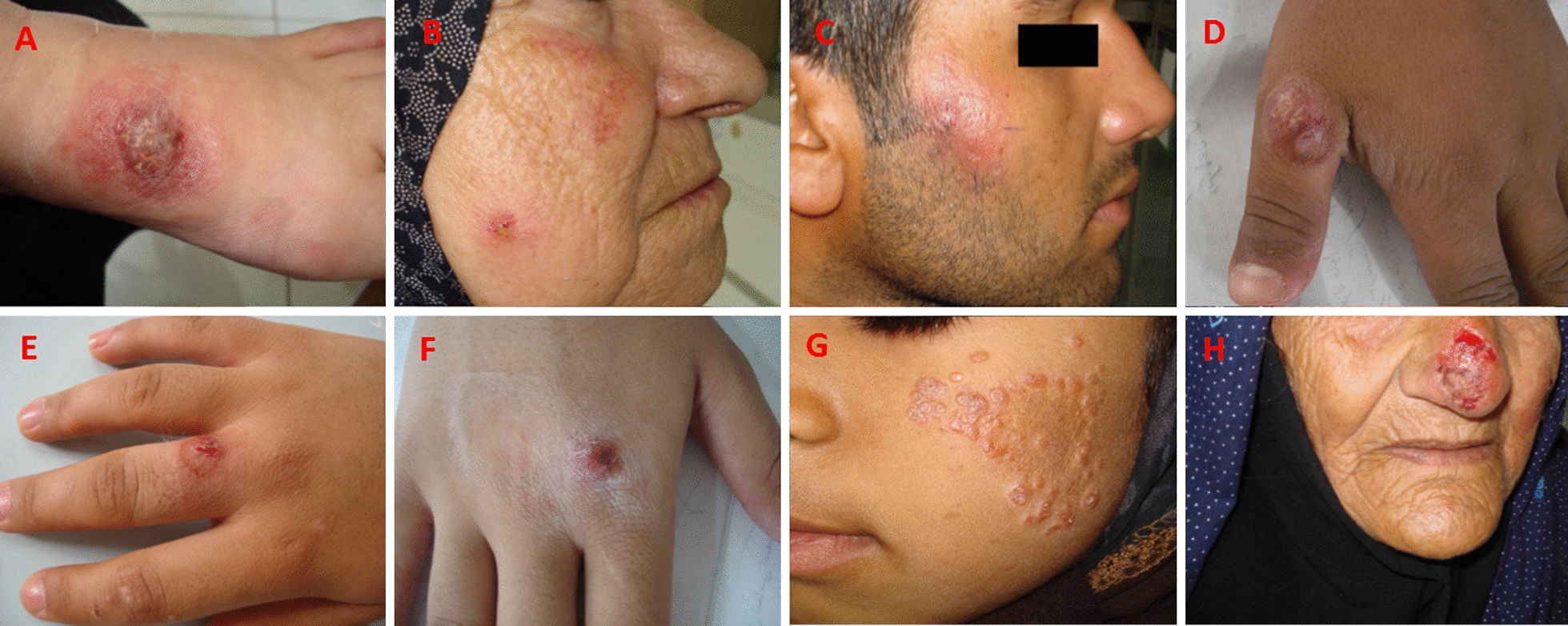
Representative pictures of anthroponotic cutaneous leishmaniasis (ACL) cases obtained from Kerman and Bam cities (**a**–**g** localized skin lesions; **h** lupoid leishmaniasis; **i** a chronic/unresponsive cutaneous lesion)

Cutaneous leishmaniasis patients were categorized into five 3-year periods to display the incidence rate of cases (Table 2 and Fig. 2a). Following the earthquake that struck Bam in 2003, the incidence rate of ACL cases significantly increased (*P* < 0.001) from 2005 to 2016 in new settlements with a sudden decline thereafter. The overall average risk of contracting the disease in peri-urban areas was 7.6 times higher in new settlements compared to the city of Kerman (Fig. 3).

**Table 2:** Baseline data indicating the new cases with anthroponic cutaneous leishmaniasis and the corresponding population in the peri-urban area compared with the city of Kerman, southeastern Iran, 2005–2019

Period (years)	Peri-urban area			City of Kerman				
	Population (no.)	Cases (no.)	Annual average (no.)	Population (no.)	Cases (no.)	Annual average (no.)	*P* value	OR
2005–2007	15,121	51	17	628,348	783	261	0.001	2.7
2008–2010	16,148	99	33	681–960	993	331	0.001	4.2
2011–2013	17.086	285	95	715,104	1041	347	0.001	10.9
2014–2016	18.078	249	83	754,992	936	312	0.001	11.2
2017–2019	18,968	165	55	796,989	930	310	0.001	7.3
Total	17,080.2	849	56.6	715,478.6	4683	312.2	0.001	7.6

All age groups were affected in peri-urban areas, but the majority of cases occurred in the < 10 year age group (*P* < 0.001) (Table 3). The overall incidence rate was 3.3 and 0.44 per 10,000 inhabitants in the peri-urban area and Kerman city, respectively. There was a significant difference (*P* < 0.001) among the new cases in the two populations. Similarly, if the age group differences were considered, the incidence rate of infection in the < 10 year olds (5.3 vs. 0.58) was significantly higher (*P* < 0.001) than it was in the > 10 years age group (2.8 vs. 0.33) per 10,000 people in the peri-urban area and the city of Kerman, respectively (Fig. 2a). The rate of infection in Afghan migrants was significantly higher than in the Iranian nationals in the peri-urban community (*P* < 0.001). Conversely, the pattern of infection in the Afghan population was substantially higher (*P* < 0.001) relative to the Iranian residents in the city of Kerman. There was no significant difference among males and females in either the peri-urban area or in Kerman city.

**Table 3:** Demographic characteristics of the patients with anthroponotic cutaneous leishmaniasis in the peri-urban area and the city of Kerman, southeastern Iran, 2005–2019

Area	Characteristics		Healthy population	No. of cases	OR (95% CL)	*P-value*
Peri-urban area	Age (year)	< 10	3586	299	2.046 (1.769–2.366)	< 0.001
		> 10	13,494	550	1	
	Gender	Male	8241	395		
		Female	8839	454	1.072 (0.933–1.23)	0.172
	Nationality	Iranian	15,342	762	1	
		Afghan	1738	87	1.008 (0.803–1.265)	0.496
Kerman City	Age	< 10	143,094	1241	1.442 (1.351–1.539)	< 0.001
		> 10	572,378	3442	1	
	Gender	Male	357,795	2337		
		Female	357,947	2346	1.003 (0.947–1.063)	0.459
	Nationality	Iranian	658,234	4238	1	
		Afghan	57,508	445	1.202 (1.09–1.326)	< 0.001
Total	Age (years)	< 10	146,680	1540	1.542 (1.453–1.635)	< 0.001
		> 10	586,142	3992	1	
	Gender	Male	366,547	2732		
		Female	366,275	2800	1.026 (0.973–1.081)	0.178
	Nationality	Iranian	673,846	5001		
		Afghan	58,976	531	1.213 (1.109–1.327)	< 0.001
	Area	Kerman	715,742	4683		
		Peri-urban	17,080	849	7.597 (7.051–8.186)	

Most of the patients were Iranian (*n* = 762, 89.7% vs. *n* = 4238, 90.5%) and to lesser extent Afghan migrants (*n* = 87, 10.3% vs. *n* = 445, 9.5%) in the peri-urban area and in the city of Kerman, respectively.

### Clinical data

The anatomical locations of the skin lesions were slightly different between the suburban area and Kerman city, but the difference was not significant. The hands were the most common site of involvement (47.5% vs. 48.1%) followed by the face (29.6% vs. 26.7%) and legs (9.1% vs. 9.7%), and the others (13.8% vs. 15.5%) were located on other parts of the body in the peri-urban area and Kerman city, respectively (Fig. 2b). Most patients in the suburban area and the city of Kerman had a single lesion (69.4% vs. 62.5%) or two lesions (19.6% vs. 21.1%), and the rest (11.0% vs. 16.4%) had ≥ 3 lesions, respectively (Fig. 2c). The average number of skin lesions was 1.5.

### Treatment outcome

Altogether, *n* = 5532 patients with CL received intralesional meglumine antimoniate along with biweekly liquid nitrogen cryotherapy or intramuscular meglumine antimoniate alone according to the national protocol. Post-treatment follow-up assessments were carried out at 3 months after completion of therapy. Of those, 84.9% vs. 85.2% were cured and 15.1% vs. 14.8% failed equally in the peri-urban area and Kerman city, respectively (Fig. 2d).

### Molecular data

**Fig. 4:**
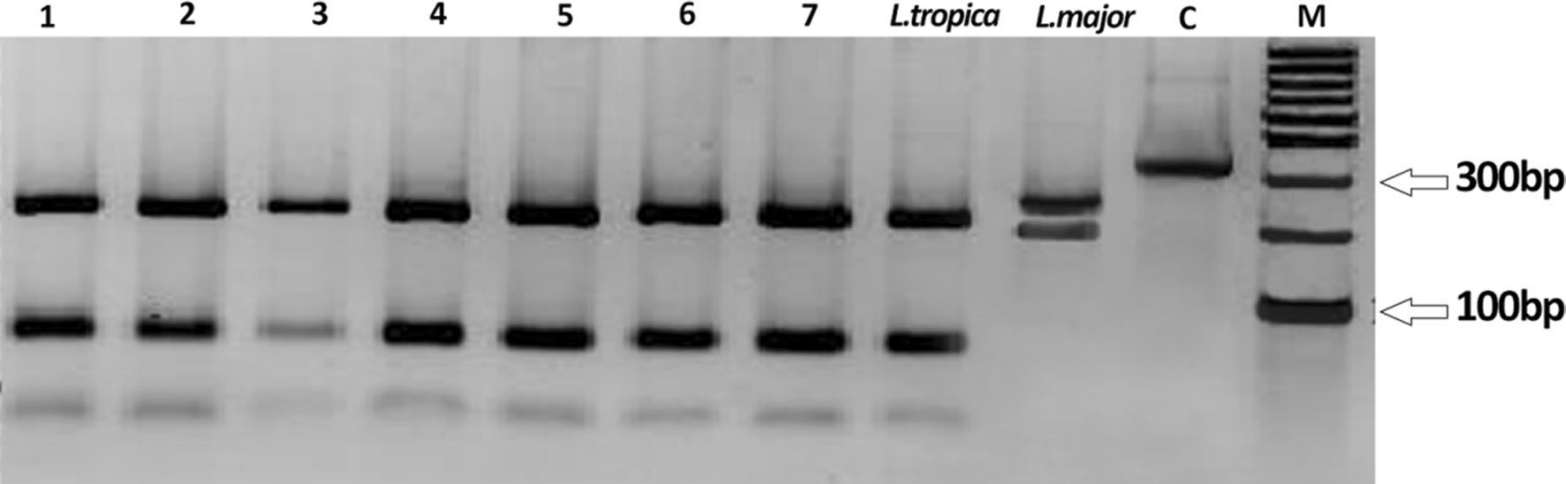
Digestion with the restriction endonuclease enzyme HaeIII of amplified ITS1 regions. Fragments were separated on 3% agarose gel electrophoresis to document differences in RFLP patterns with two bands of 185 and 58 bp for *Leishmania tropica*. Positive control samples (*L. tropica and L. major*), no enzymatic digestion isolates 330 bp (C), and representative clinical samples of anthroponotic cutaneous leishmaniasis (ACL) cases obtained from peri-urban areas, southeastern Iran [1–7]

The ITS1 PCR amplified a 330-bp gene fragment on 2% agarose gel electrophoresis. All 50 randomly selected clinical isolates were confirmed to be purely *L. tropica*, the etiological parasite of urban CL in the Old World (Fig. 4).

### Phylogenetic profile

**Fig. 5:**
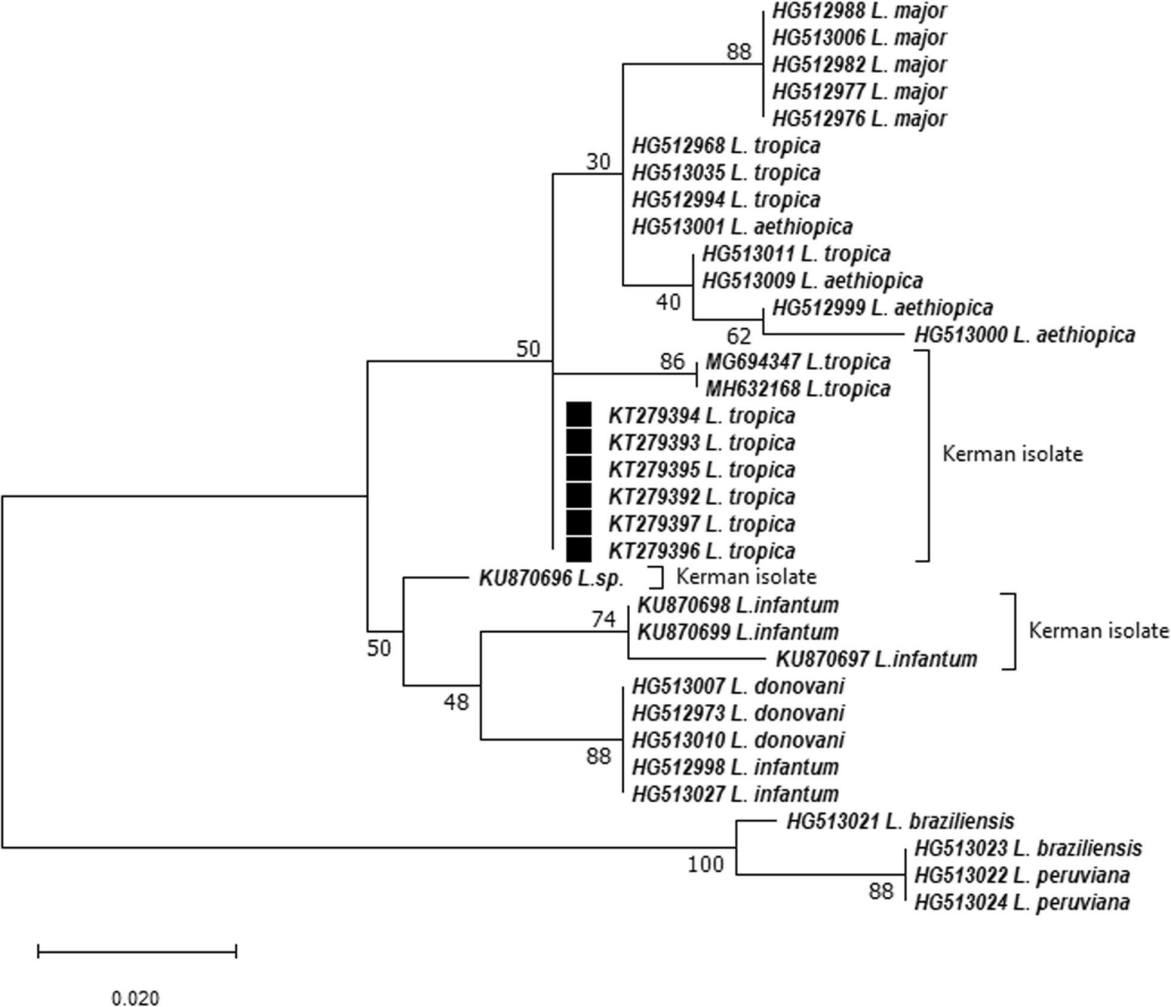
Maximum likelihood phylogenetic tree of *Leishmania tropica* isolated from the peri-urban area, southeastern Iran

After confirmation through PCR-RFLP, 18 sequenced amplicons of the 7SL RNA gene were used for phylogenetic analysis. Randomly selected nucleotide sequences were clustered into six variants and submitted to the GenBank database (KT279392-KT279397) (Fig. 5).

The numbers presented at the internodes indicate bootstrap support percentages (1000 replicates). To identify the type of isolated strains, at least three reference sequences were selected from each *Leishmania* species, using the list of WHO *Leishmania* reference strains. Accession numbers of isolated clinical samples in this study were also noted at the tips of the corresponding branches. All Kerman isolates in the current investigation were confirmed to belong to the *L. tropica* complex; however, a significant phylogenetic distance was observed with previous records of the province (MG694382 and MH632168). The accession number and species name of the sequences are given at the tips of the consistent branches, and the dissimilarity scale is portrayed at the bottom left corner in replacements per nucleotide.

## Discussion

In this study, we described a long-lasting emerging epidemic of ACL in peri-urban areas of the city of Kerman. The average risk of contracting ACL was significantly higher in new settlements than in Kerman city between 2005 and 2019. Kerman is a geographically vast, ecologically diverse, and topographically disaster-prone eco-region, and CL is a poverty-driven disease. Therefore, such a combination provides various ecological and biological risk factors in favor of emerging CL epidemics. This disease is considered the most emerging and re-emerging disease, which has recently increased to a hyperendemic level in some countries and extended its traditional territories to new foci [5, 46]. This is mainly due to the coexistence of complex determinants reinforcing suitable conditions for the proliferation of female *Ph. sergenti* flies and transmission of the causative agent, *L. tropica,* from human to human.

Furthermore, several massive earthquakes have stricken cities in the province, including Bam, Zarand, and Ravar counties, leaving behind approximately 40,000 survivors, 35,000 injured, and 60,000 homeless people. Moreover, the province has experienced severe droughts and rapid urbanization; therefore, varying arrays of relevant risk factors have been provided, negatively affecting health and resulting in multiple social, economic, and environmental consequences [41, 42, 47–49]. The last 2 decades have witnessed a massive migration from the peripheral counties and communities to the peri-urban areas of Kerman city, mostly attributed to prolonged droughts and natural disasters. Migrants reside in peri-urban shantytowns, built-in proximity to Kerman city, where the land is inexpensive. Still, they have to grapple with severe socioeconomic conditions and poor health services.

Population movements are strongly associated with the spread of neglected tropical diseases, particularly leishmaniasis, and are often exacerbated by poor medical services and insufficient sanitary infrastructures. Leishmaniasis is an example of the consequences engendered by population mobility. Based on the International Organization for Migration, > 200 million people are reported to be global migrants, and in about 52 countries, approximately 30 million are displaced within their own countries as the outcome of civil war and conflict, not to mention those recently driven away from their homes by famines, droughts, floods, and various other natural and human-made disasters [50].

Iran is home to diverse ethnic groups and has recently faced multiple health concerns. It is estimated that approximately 3 million Afghan people live in Iran because of the prolonged and ongoing civil war, violence, and political and socioeconomic instability in their homeland. Most of these migrants intended to engage in different professions temporarily but, for the most part, ultimately became permanent residents of the country. Kerman province has fertile and cultivable land and is among the three major agricultural provinces where many seasonal and permanent laborers, either indigenous or Afghan (~ 300,000) migrants, work in gardens and crop fields. However, different typographies and patterns of residency and mobility exist in the province; generally, most migrants are poor, and their living and working conditions have created a suitable environment for CL transmission.

Moreover, the cross-border movement from neighboring countries is a major challenge for a comprehensive CL control program. Iran has one of the longest borders [2, 6] with neighboring countries in Western Asia. The migration of many people from Afghanistan, Pakistan, and Iraq to Iran, where the conditions are suitable for the spread of the parasite, vector, and reservoir, could further propagate the disease. When a country shares long borders with multiple countries, cross-border movements are a common problem. A remarkable example of such a situation is the Syrian migrant crisis, which is the largest refugee and dislocation crisis of the century. Approximately 6 million Syrians are refugees, and about 6 million people are displaced within Syria. A population of over 12 million in Syria requires charitable support. At least 50% of the people involved in the Syrian refugee crisis are children. Health clinics and hospitals, financial institutions, schools, universities, and sanitation systems have been seriously hampered or demolished. Once busy markets have been devastated. War has destroyed the infrastructures, businesses, and social ties that bind people to their community. This ongoing civil war has aggravated old endemic CL foci to an epidemic state. It has increased the number of new cases of CL and the growth of the disease to new foci nationally and abroad [51].

The past few decades of civil conflict, war, and social unrest have hindered attempts to control ACL in Afghanistan. As a result, this country has been experiencing a prolonged epidemic [14]. Kabul has recently been reported to be the world’s most extensive ACL endemic focus [50, 51]. The persistence of the epidemic was associated with the continuous flow of immigrants, providing a constant reservoir of non-immune individuals [14]. Moreover, in Pakistan, refugees transmitted ACL into previously unaffected areas where a large urban CL outbreak in the refugee camp was recorded [52]. The primary mechanism is the displacement of the population into new settlements, where they become exposed to the source of infection where the biological vectors breed; either the migrating people arrive into new endemic areas and contract the disease through female sandfly bites or they carry the ACL infection as the main reservoir host and transmit the parasite to the unexposed population. Historical examples are the intrusion of ACL into different rural areas within this province following the earthquake, population flow, rapid unplanned urbanization, and fast and extensive road building in the last decade [31, 32, 35].

Leishmaniasis is a climate-sensitive complication, and fluctuations in precipitation, humidity, and temperature have a profound effect on the ecology, spread, and biodiversity of sandflies and reservoir hosts, impacting their distribution and hence facilitating the transmission of the causative parasite. During the last decades, profound droughts, devastating earthquakes, and frequent floods have had severe direct and indirect detrimental effects on groundwaters, rangelands, and agricultural crop productions. As a result of such alterations in Kerman province, many people (65,000 citizens) have lost their jobs and abandoned their villages. Such social and economic forces were the primary driverss that forced them to leave their occupations, which led to massive displacement and large-scale migration of the population to various cities and new peri-urban areas with high potential for ACL transmission [53].

Generally, the problem of peri-urban residents has been overlooked in the mainstream delivery of not only PHC services globally [54] but also essentials for standards of living. The peri-urban settlement adjacent to the city of Kerman has become a newly emerging ACL focus and an important concern in the epidemiology of ACL. *Ph. sergenti* has become widely dispersed as the principal vector of ACL in this area [55, 56]. In Iran, ACL is caused by *L. tropica*, which is mainly limited to humans [26], although sporadic cases of dogs being infected with ACL have been reported in this emerging focus [57, 58]. The actual role of dogs in the life-cycle of the parasite is not well known. It is assumed that they might be implicated in the urban life-cycle and play a secondary role in transmitting the *L. tropica* parasite [59].

The incidence of infection was analyzed. Although *L. tropica* infected all age groups, most cases occurred in children younger than 10 years in both the pre-urban area and Kerman city (5.3 vs. 0.58 per 10,000 population, respectively). These results are in agreement with the previous findings reported by others [60, 61]. Generally, children are less disciplined about covering themselves at night; they spend more time outdoors and hence are more frequently exposed to female sandfly bites than other people. Furthermore, children are at a higher risk of clinical disease due to their immature immune systems and are most easily infected by CL [62]. Peri-urban settlements under poor socioeconomic conditions increase the risk of CL. Impoverished domestic sanitary conditions and unsuitable housing environments, including open sewage or lack of solid waste management, might enhance sandfly resting and breeding sites and facilitate the transmission of the disease to humans. The lack of personal protection measures, civil unrest, disasters, and economically driven migration bring non-immune humans into contact with infected sandflies [63]. Public investment in management and control strategies would reduce the disease burden and help alleviate poverty [64].

Skin lesions can be either single or multiple on the hands, face, or other parts of the body. The exact reason for sizeable solitary lesions on the hands as the predominant involvement site is not well understood. This could be due to various environmental and behavioral factors associated with human habitation, parasites, and vector species. These results are also consistent with previous findings reported from these areas [35, 41]. This finding is consistent with the results found in the southeastern part of Iran [35, 65]. In contrast, in the previous report [60], the face was the most common site of involvement, followed by the hands and other body parts.

In Iran, meglumine antimoniate is the drug of choice for the treatment of ACL and is used either by intralesional administration in combination with cryotherapy or systemically alone based on the national guideline. The efficacy of both treatment regimens was in agreement with those reported from the southeast in the pre-urban area and also in the city of Kerman. Like in the city of Kerman, CL is passively detected in residents in the peri-urban area. Those who are suspected of being infected are referred to the CL management unit at Dadbin Clinic to be diagnosed and treated accordingly. Treatment failure is a common occurrence in Iran, particularly in endemic foci where ACL is prevalent, ranging between 10% and 15 % [41].

One of the secondary objectives of the current study was to identify the causative parasite and analyze the genetic variability of *L. tropica* isolates obtained from patients with ACL. Among all Leishmania genus species, *L. tropica* shows a high level of genetic polymorphism [43]. Applying the highly sensitive and powerful diagnostic technique directly from clinical samples could be an important tool for identifying the species and determining the disease's geographical distribution. This could be a fundamental approach to planning future control programs [4]. In this study, the implementation of PCR-RFLP analyses of the ribosomal ITS1 amplicons followed by partial 7SL RNA sequencing revealed different heterogeneity patterns compared to the national and international isolates. Overall, six genetic variants were detected among the sequenced isolates.

The existence of different *L. tropica* genotypes in the city and peri-urban areas highlights the high levels of heterogeneity of this species within the study area in the province of Kerman. These variants were closely related and clustered together with other previous isolates obtained from Bam and Kerman counties [30, 35, 66]. The overall pattern of ACL infection indicates that the etiological agent of ACL is propagated and transmitted from person to person from dissimilar clones as reflected by the complexity of the migrants’ backgrounds. This finding also showed that different haplotypes are presumably associated with various clinical features that respond differently to conventional drugs [41, 61]. Efforts to compare the phylogenetic results with clinical isolates from Afghanistan were inconclusive as no 7SL RNA gene analysis was available in the literature.

Several gene regions, including ITS1, 7SLRNA, and Hsp70 sequences, have been used to compare the genetic diversity among non-healed and healed isolates of ACL cases in the main foci in Iran [66]. Among the three gene regions, 7SLRNA and Hsp70 were genetically identical, while the ITS1 region showed nucleotide heterogeneity patterns between the unresponsive and responsive forms. Hence, the ITS1 genetic marker could serve to inspire further future investigations on the function of these variations among different non-healing clinical forms of patients with ACL. The VL caused by *L. tropica* was also revealed to be genetically heterogeneous [67] and to play a key role in the generation of drug-resistant leishmaniasis recidivans in the southeastern part of Iran [68]. *Leishmania tropica* isolates in the southeast (Kerman and Bam counties) represented different genetic polymorphisms using PCR-RFLP of the kinetoplast DNA (kDNA) [67].

Toz and colleagues have already used real-time PCR as a powerful and prevailing tool to identify clinical samples [69]. Giemsa-stained slides are valuable sources to be used for PCR for the identification of *Leishmania* isolates to the species level. In the present study, all isolates were *L. tropica*, the causative agent of urban CL in the Old World. Previous studies also employed ITS1 PCR on clinical smear preparations for *Leishmania* species identification [60, 70]. Such a molecular tool provides fundamental approaches to understanding the epidemiological characteristics in order to select the disease’s proper treatment modality [71].

Population displacement and migration are rapidly becoming issues of public health concern; they require particular policy attention at many levels in developing countries. The provision of all the essentials to such unprepared communities is not possible. Problems of peri-urban settlements have been somewhat overlooked in the process of service delivery. A robust commitment to a multilateral approach is crucial to making improvements in this area. This will require decisive coordinated actions through all governmental factions, including policymakers, health professionals, senior researchers, and non-governmental organizations (NGOs).

There has been no study similar to this work to the best of our knowledge, and it is the largest observation carried out on a vulnerable peri-urban community where population mobilization is directly associated with CL. This investigation’s major strength is assessing a long-lasting emerging CL epidemic for 15 years, with many cases. This achievement is mainly due to the well-coordinated work, carried out by expert academic members, linked with a team of experienced health personnel and staff. This study has also drawn attention to the importance of population mobility to CL as a major neglected tropical disease in a highly endemic country.

The current investigation had many limitations. First, although CL is a notifiable disease in Iran, cases are only detected through passive strategies. Implementation of active case-finding approaches could help assess the actual burden of the disease for planning future control programs. Second, due to the complexity of the population and the presence of so many environmental and socio-cultural determinants, it was not feasible to provide comprehensive advice. Third, methodologically, such studies are complex and challenging as they assess interactions of health and population movements over large distances and relatively long periods. This requires large multicenter and multidisciplinary projects. Consequently, multiple studies exploring the local cases and challenges to health care services related to mobile populations are essential.

In conclusion, the movement of populations and the establishment of new settlements in peri-urban areas in close association with endemic areas are major risk factors and are directly linked to the occurrence of CL, either contributing to exposure of the vulnerable and at-risk population to new risks or leading to the introduction of the causative agent into new areas. The risk of contracting ACL is further exacerbated by poor socioeconomic backgrounds and lack of adequate health infrastructures in the new settlement. Therefore, public health surveillance systems and clinical practitioners should know the new settlement’s basic needs. Furthermore, promoting public awareness, directly or by social media, mainly in high-risk areas where the disease is notifiable, active and passive case detection strategies, early diagnosis, and effective treatment modalities could help control the disease. Besides, public investment in infrastructures, management, and control strategies will reduce the disease burden and alleviate poverty.

## Data Availability

The datasets used and/or analyzed during the current study are available from the corresponding author on reasonable request.

## Abbreviations

CL: Cutaneous leishmaniasis
ACL: Anthroponotic cutaneous leishmaniasis
ITS1: Internal transcribed spacer1
PCR: Polymerase chain reaction
RFLP: Restriction fragment length polymorphism
VL: Visceral leishmaniasis
MCL: Mucocutaneous leishmaniasis
ZCL: Zoonotic cutaneous leishmaniasis
IRB: Institutional review board
